# Stem Cell Paracrine Signaling for Treatment of Premature Ovarian Insufficiency

**DOI:** 10.3389/fendo.2020.626322

**Published:** 2021-02-24

**Authors:** Alba M. Polonio, Juan A. García-Velasco, Sonia Herraiz

**Affiliations:** ^1^ IVI Foundation, Insituto de Investigación Sanitaria La Fe, Valencia, Spain; ^2^ IVI RMA, Madrid, Spain; ^3^ Department of Obstetrics and Gynecology, Rey Juan Carlos University, Madrid, Spain

**Keywords:** follicular rescue, ovarian rejuvenation, premature ovarian insufficiency, stem cells, autologous stem cell ovarian transplant, mobilization

## Abstract

Premature ovarian insufficiency is a common disorder affecting young women and represents the worst-case ovarian scenario due to the substantial impact on the reproductive lifespan of these patients. Due to the complexity of this condition, which is not fully understood, non-effective treatments have yet been established for these patients. Different experimental approaches are being explored and strategies based on stem cells deserve special attention. The regenerative and immunomodulatory properties of stem cells have been successfully tested in different tissues, including ovary. Numerous works point out to the efficacy of stem cells in POI treatment, and a wide range of clinical trials have been developed in order to prove safety and effectiveness of stem cells therapy—in diminished ovarian reserve and POI women. The main purpose of this review is to describe the state of the art of the treatment of POI involving stem cells, especially those that use mobilization of stem cells or paracrine signaling.

## Introduction

In humans, oocyte development begins during fetal life and follicle pool reaches its maximum at 16/20 weeks of fetal development (1). Follicular decline is initiated before birth, so that, at the time of delivery, only about 1 million of follicles remain in the ovary of the baby. By the time of menarche, each ovary contains about 400,000 follicles and ovarian reserve continues decreasing as women age (2). Thereby, the decline in oocyte quantity and quality during women reproductive life is a physiological process; however, in some women, ovary deterioration occurs in an abrupt way and they become prematurely infertile.

Primary Ovarian Insufficiency (POI), also known as Premature Ovarian Failure (POF) or premature menopause, is a reproductive disorder, characterized by oligo-amenorrhea and high levels of serum FSH, leading to a cessation of ovarian function before the age of 40 (3). This condition affects 1% of women under the age of 40 years, and 1 out of 250 women under the age of 35 years (4).

POI is characterized by a hypergonadotropic hypogonadism state, which can be diagnosed by a triad of features in a woman under the age of 40: (a) postmenopausal levels of follicle-stimulating hormone (FSH) (>40 UI/L in two different samples taken separately in the time), (b) 4 or more months of amenorrhea, and (c) decreased estradiol serum concentrations (3). These patients present low AMH serum levels and a low antral follicle count (AFC).

This condition represents a dramatic scenario, as ovarian dysfunction leads to female infertility in POI patients. Laparoscopy shows a lack of follicle development in POI patients and dysfunctional ovaries lead to estrogen deficiency. The uterus and vaginal mucosa undergo atrophy, which is very often associated with dyspareunia (5, 6). In addition, POI involves menopausal syndrome, which may include hot flushes, night sweats, heart palpitations, insomnia, or headaches. Moreover, POI is associated with long-term negative consequences in female health, such as an increased risk of immunological disorders, cardiovascular diseases, and osteoporosis (7).

It should be noted that 5–10% of women with POI might have spontaneous follicular development, menses resumption, or spontaneous pregnancies, especially during the first year after diagnosis (8). This could be due to the fact that ovarian biopsies from POI patients revealed that up to 9% of women have plenty primordial follicles, and 30% have some primordial follicles (5, 9). However, ovulation is unpredictable and most women with POI have a low chance of pregnancy (5, 6, 10).

There are limited options for POI patients, whose treatment may be oriented to reduce the impact of endocrine dysfunction—by means of therapy hormone replacement—and/or to overcome infertility. None of these strategies are absent from limitations. On the one hand, hormone replacement therapy has been associated with an increased risk of reproductive cancer (11–15). On the other hand, the treatment of POI-associated infertility by reproductive techniques with autologous gametes represents a major challenge in reproductive medicine, and usually involves prolonged protocols and inconsistent clinical outcomes. Despite the intensive effort to develop variants of stimulation protocols to improve reproductive outcomes in poor responder patients, intrinsic characteristics of POI patients make its management even more difficult; probably, because of the absence of antral follicles responding to stimulation. To date, there is no enough evidence to recommend most of these strategies in order to improve pregnancy rates in POF patients (16, 17). In most cases, the only options for these patients to achieve desired motherhood is oocyte donation or adoption, which are not always accepted due to ethical, cultural, or religious issues. Thus, because of the complexity of this disorder, a standard and effective treatment has not yet been established; but an active management of these patients and an intensive search for new strategies may open new doors for them.

## Etiology

**Figure 1:**
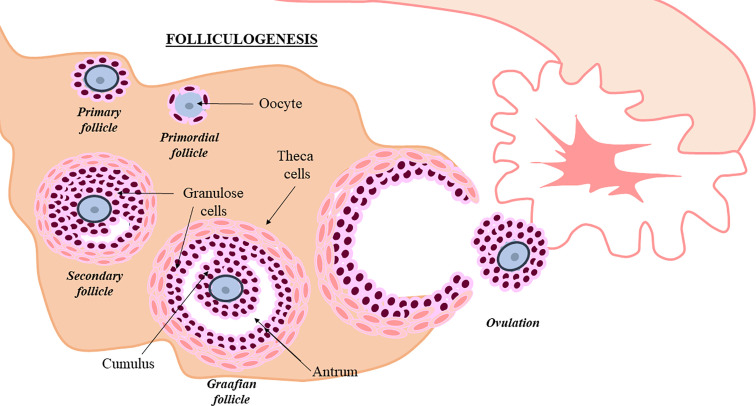
Follicullogenis. Granulosa and theca cells assist oocyte progression towards ovulation (10). Follicle development is shown in the picture.

The human ovary contains a limited and non-renewable pool of quiescent follicles determined at birth. Folliculogenesis is a complex process that should be extremely regulated. During this process, granulose and theca cells assist the oocyte, in order to promote primordial follicle development towards antral stage and ovulation (10) (**Figure 1**). Intraovarian mechanisms activate a small number of primordial follicles (≈1,000/month) and although most underwent atresia, a few of them achieved the advanced maturation stage before ovulation (18). Follicle depletion occurs at menopause when less than 1,000 quiescent follicles remain (19). In POI, this process is altered. It is suggested that follicular dysfunction and altered follicle depletion may underlie POI (20). Although scientific knowledge is limited about factors controlling oocyte pool and the cause of POI is not yet completely understood, different factors could alter follicle maintenance and development. In fact, POI can appear spontaneously or induced by different factors (21).

The most common cause of POI are oncologic treatments with high doses of chemo- and radiotherapy (22). The increased survival rates (>80%) of oncologic patients associate a growing percentage of young women facing gonadotoxic side effects of cancer therapies without having accomplished their reproductive project. Deleterious effects on the ovary depend on the age of the patients—the risk to develop POI after cancer therapy increases with the age—the dosage and type of toxic agent (having the alkylating agents the highest risk for developing POI). A main mechanism of chemotherapy-induced ovarian failure is based on the damage induced to DNA of primordial follicles, leading to apoptosis, and promoting a massive activation of follicles followed by atresia and elimination. This follicular depletion also associates an impairment of ovarian vascularization, fibrosis, or interrupting cross talk communication between follicular cells (23, 24), leading to a cessation of ovarian function

Iatrogenic factors, such as laparoscopy, ovarian drilling, or surgery for ovarian endometriosis or cysts, may also lead to POI (25). Others environmental factors such as viral infections or pollutants can result in POI, although the real incidence of these cases is not clear (26, 27).

Genetic defects, including X chromosome aneuploidies (Turner syndrome, trisomy X) (28–30), structural X chromosome anomalies (isochromosome, deletions, inversions, duplications) (31), mutations or premutations of X linked genes (Fragile X syndrome) (31), and single mutations in genes related to reproductive function (FSH receptor, LH receptor, inhibin, galactosaemia) constitute another cause of POI (32). Enzymatic deficiencies in the steroidogenesis pathway could also lead to POI (22).

Finally, autoimmune mechanisms are involved in pathogenesis of more than a 4% of POI cases (33) and autoimmune disorders such as myasthenia gravis, celiac disease, vitiligo, lupus, Addison’s disease, or autoimmune polyglandular syndrome, have been seen in a percentage of women diagnosed with POI (22, 34). In these patients, immune alterations including an increase in CD4+ T cells and B cells, macrophage and dendrite cells disorders, lymphocytes oophoritis, and inappropriate expression of class II MHC antigens by granulose cells have been found (35–37). In fact, anti-ovary antibodies—with several targets—have been detected in 50% of unexplained infertile patients and several studies report that the presence of autoantibodies increases the risk to develop POI in patients with autoimmune disease (22, 34).

However, most cases of POI are idiopathic (22), which promote further investigations to utterly understand this entity, in order to explore new strategies to solve it. Even in cases with a diagnosed cause, the diversity of disorders associated with POF indicates the heterogeneity of this entity. This fact underlines the need not only to develop different strategies to improve clinical management of these patients, but also the importance of the selection of the right population of POI patients, who can benefit from each approach.

## Novel Strategies for POI Management

Recent research has focused the attention on the residual quiescent pool of follicles that remain even when the ovary loses their ability to ovulate and function. Based on the successful protocol of *in vitro* activation (IVA) of primordial dormant follicles, Kamawura et al. was the first group exploring the combination of IVA with mechanical ovarian fragmentation, to inhibit the Hippo pathway, in menopausal women (38). Additional research has been developed to improve the success of the technique and to design a less invasive procedure, named as one step IVA or ovarian fragmentation for follicular activation (OFFA). The technique consists in a unique surgery for ovarian cortex retrieval, followed by mechanical fragmentation into small pieces and transplant into an ovarian grafting site. By means of this strategy, 10 premenopausal women have achieved a pregnancy (39–43) as well as several poor ovarian responders (POR) (44). OFFA pursues not only fertility recovery but also endocrine function and as avoids ovarian cryopreservation, the main concern of ischemia-associated follicle death is also overcome.

Artificial ovary is also a promising alternative that will be used to *in vitro* growth and maturation approaches or to improve ovarian transplant in the future. Although it has been applied successfully in animal models, its efficacy and safety have to be proved before it becomes a reality for patients (45).

Another strategy, closely related to the previous one, is the generation of artificial gametes in patients who are not able to produce functional gametes. Artificial gametes could be generated from induced pluripotent stem cells (iPSCs), embryonic stem cells (ESCs) derived from blastocysts inner cell mass, or the putative germline stem cells (GSCs) (45). In animals, artificial gametes have been generated from GSCs (46), ESCs (47), and iPSCs (48, 49). The implementation of this technique in animal preclinical models has even achieved the birth of viable offspring (48, 49). The main limitation of the technique—apart from multiple ethical concerns—is the low efficacy of differentiation. In humans, artificial oocyte-like cells have been developed from ESCs (50) and GSCs (46) and the successful fertilization of artificial oocyte-like cells has been reported (51). However, development potential and human offspring from artificial gametes is to date far from reality (52). In relation to this strategy, transplantation of ovarian granulosa-like cells derived from human IPSCs has been reported to repair ovarian niche and to promote follicular development in POF mice (53).

Different studies have described a lower telomere length and telomerase activity in POF patients (54–56). Concerning this pathway, the reactivation of telomerase—which maintains telomere length—has been described to resume fertility in telomerase-deficient mice, which present impaired fertility (57, 58), opening a new future possibility to ovarian rejuvenation by means of telomerase reactivation (59–61).

Based on the autoimmune dysregulation associated to a wide percentage of POI cases, together with the close association of fibrosis with ovarian failure, some authors suggested the possible effect of immunomodulating therapy for ovarian function recovery in POF patients, especially in those with autoimmune-related POF (34, 62, 63). In fact, use of the anti-inflammatory and antioxidant properties of several agents has been proved to improve ovarian function in a POI mice model (62, 64).

## Stem Cell–Based Approaches

### Preclinical Studies

Most of these strategies highlighted the relevance of the ovarian niche as a key parameter to promote an adequate follicle development in order to restore ovarian function. Following this idea, one of the most promising strategies pursues the regeneration of ovarian niche using Stem Cells (SCs) in order to promote development of remaining follicles within the ovary.

With the rise of regenerative medicine, different types of SCs have been tested for follicular rescue and regeneration of the ovarian niche. Among them, **mesenchymal stem cells (MSCs)** have been the most widely used for these strategies. MSCs are a population of SCs that can be derived from different adult tissues, and that have proliferative, self-renewal and differentiation to different lineages properties (65). Different studies in animal models with different degrees of ovarian damage describe the ability of MSCs to restore ovarian function in these animals.

**Mesenchymal stem cells from human and murine amniotic fluid** have shown the ability to survive and proliferate in the ovary and to rescue short-term fertility of mice with chemotherapy (QT)-induced POF after injection into the ovarian artery (66, 67). Wang et al. report the ability of amniotic epithelial stem cells (AESCs) to infiltrate the damaged ovary after injection into the tail of mice with POF (68), leading to the recovery of folliculogenesis and differentiation towards granulosa cells (68, 69). Ding et al. show the recovery of the follicle pool in all its developmental stages and hormonal restoration after the tail injection of both AESCs and amniotic mesenchymal stem cells (AMSCs) in mice with different degrees of ovarian failure induced by chemotherapy (70). Although AESCs seem to show less immunological rejection, AMSCs show a higher efficacy in the recovery of ovarian function, especially in the most drastic cases of POF (70). Ling et al. evaluate the improvement of the treatment with human AMSCs by pretreating them with low intensity pulsed ultrasound (LIPUS). Both LIPUS-pretreated AMSCs and non-pretreated AMSCs have been reported to increase reproductive organ weights, reduce granulosa cells (GCs) apoptosis and ovarian inflammation, and improve ovarian function in POI rats (71).

MSCs can also be obtained from the umbilical cord. The injection of **umbilical cord mesenchymal stem cells (UCMSCs)** into the tail vein allows improvement of the ovarian structure and ovarian function—at the hormonal and follicular level—in mice with POF induced by QT and in rats with natural ovarian aging. GCs apoptosis reduction and cytokines secretion leading by UCMSCs are proposed as possible mechanisms of action (72–75). Zhu et al. report that the recovery of ovarian function and fertility after UCMSCs transplantation occurs sooner when UCMSCs are injected directly into the ovarian artery (76). It has also been reported a long-term survival of UCMSCs in the rat ovary after the transplant (73) and stabilization of the ovarian epithelium by these SCs (74). Both human UCMSCs and AMSCs interventions restore ovarian morphology elasticity and toughness, and involve a slight recovery of ovarian function in QT damaged ovaries (77).

Menstrual blood is another possible source of MSCs. **Human menstrual blood-derived stem cells (MenSCs)** are endometrial MSCs, which have also been used for the treatment of POI. Thus, the ability of these cells to migrate to the ovary has been reported, and ovary infiltration by MenSCs is followed by hormone levels restoration and follicular count increase in mice with QT-induced POF (78–80), as well as the restoration of fertility in these mice (78). The reduction of both fibrosis and apoptosis through cytokine secretion has been suggested as possible mechanisms to restore ovarian function by MenSCs (78, 79). Feng et al. also described a possible role of these cells in the regulation of folliculogenesis (80). Recovery of ovarian function has been also achieved by injecting only the culture medium of MenSCs, which reinforces the idea of paracrine action of these cells (79). Thus, MesSCs would represent an interesting alternative due to the possibility of non-invasive collection. Nevertheless, most of POI patients present amenorrhea or oligomenorrhea, which reduces the application of this strategy.

**Adipose tissue-derived mesenchymal stem cells (ADMSCs)** have also shown the ability to restore ovarian function, increasing the number of follicles after injection into the ovary in QT induced POF mice and rats (81–83). An improvement in estradiol serum levels and an increase in the gestation rate have been reported after ADMSCs transplant (82, 83). Fouad et al. report a therapeutic efficacy of both human AMSCs and ADSCs, but with a greater efficacy of the former, which achieve not only an increase in estrogen levels, but also a decrease in FSH levels in mice with POF (83). ADMSCs have been suggested to produce cytokines and reduce apoptosis in GCs (81). However, a low long-term permanence of these cells in the ovary has been reported (81). The transplantation of soluble collagen with ADSCs improves the short-term permanence of ADSCs in the ovaries and contributes to the restoration of ovarian function (82). Takehara et al. also describe a restoration of ovarian function after local injection of male ADMSCs into female mice with QT induced POF, and note that the Y chromosome only appears in theca cells and not inside the follicle. They report an increase in secreted levels of vascular endothelial growth factor (VEGF), insulin-like growth factor (IGF), and hepatocyte growth factor (HGF) (84).

Although promising results obtained for ovarian rescue with different types of MSC, their clinical application requires cell culture procedures to reach clinically relevant cell numbers for transplant. This represents a main limitation for use, as the accumulation of genomic and epigenomic alterations and degeneration in progenitor potency in human SCs have been associated to cell expansion procedures (85).

**Bone marrow derived stem cells (BMDSCs)** present an interesting alternative for transplantation in women with POI (**Table 1**). The possibility of obtaining a large number of BMDSCs, from an autologous source, by means of well-established clinical protocols—used for BM transplant after QT—makes them a valuable candidate (96). This possibility lets us avoid cell expansion steps, which is associated with genetic instability (97). In 2007, Lee et al. described fertility rescue in a QT-induced POF rat model after injection into the tail of BMDSCs derived from another rat. All the offspring belonged genetically to the recipient rat, although donor immature oocytes were described (86). Fu el al., in 2008 describe the improvement of ovarian function after the local injection in the ovary of BMDSCs derived from human in mouse with POF, possibly mediated by the reduction of apoptosis in GCs (87). The ovarian function improvement with BMDSCs has also been proved by prolongation of reproductive potential in mice beyond the common age of reproductive senescence with monthly infusions of BMDSCs from young mice (88). The increase in the number of follicles and the size of the reproductive organs, as well as the restoration of hormonal levels have been described in genetically generated POF mice, and QT induced POF mice, rats, and rabbits after heterologous distal BMDSCs transplantation (90, 91, 98). In 2014, Liu et al. describe for the first time the ability of BMDSCs to migrate into POF-damaged rat’s ovary, where they were not distributed in a uniform manner. Their presence was described to be higher in the medulla than in the cortex. Following BMDSCs transplantation, increased estradiol levels and antral follicle count were reported (89). Different studies describe not only the regenerative potential of BMDSCs, but also a protective property, with a reduction of apoptosis in ovarian cells and lower germ cell DNA damage when combining chemotherapy with injection of BMDSCs (99, 100). Heterologous transplant of BMDSCs also shows the potential to reverse the hormonal dysfunction caused by QT in mice and an increase in the number of healthy follicles has been showed after BMDSCs tail vein injection (92). The ability of autologous BMDSCs transplant to restore fertility and shorten estrous cycles has also been reported after SCs injection in the ovarian artery in a QT-induced POF mouse model (93). After that, the ability of human BMDSCs transplant to increase ovarian weight and follicular count, and to improve pregnancy rate after injection into the ovarian artery has been shown in a mouse model with QT-induced POF (94). Herraiz et al. described the restoration of fertility in a mouse model with ovarian damage after transplantation of human BMDSCs, with an increase in the number of preovulatory follicles, MII oocytes, spontaneous pregnancy rate, and number of healthy offspring (95). Furthermore, this study shows for the first time, the ability of BMDSCs to migrate towards the follicles and vessels in human tissue POR women xenografted into immunodeficient mice, promoting follicular development, ovarian local vascularization, estradiol secretion, and reducing apoptosis (95).

**Table 1:** Animal studies involving bone marrow stem cell in POI/POF models.

Regenerative factor	Study population	Administration method	Main findings	Reference
Murine BMDSC	QT induced POF mice	IV tail injection	Fertility rescue. All the offspring come genetically from the recipient, but oocytes from the donor are described.	Lee et al. (86)
Murine BMDSC	QT induced POF rats	Direct injection in ovary	Ovarian function improvement. GCs apoptosis decrease.	Fu et al. (87)
Murine BMDSC	Natural aged mice	IV tail injection	Prolongation of reproductive potential	Selesniemi et al. (88)
Murine BMDSC	QT induced POF rats	IV tail injection	Ability of BMDSC to infiltrate damaged rat ovaries. E2 and AFC increase.	Liu et al. (89)
Murine BMDSC	FSH knockout mice	IV tail injection	Follicle count and mature follicles increased. Hormonal levels restoration. Size of reproductive organs increase.	Ghadami et al. (90)
Rabbit BMDSC	QT-induced POF Rabbit	IV ear injection	E2 levels and follicle count increase and FHS levels decrease.	Abd-Allah et al. (91)
Murine BMDSC	QT-induced POF mice	IV tail injection	Hormonal levels rescue. Healthy follicles increase and apoptosis decrease.	Bao et al. (92)
Murine BMDSC	QT-induced POF mice	Direct ovarian infusion	Fertility restoration and shortening of estrous cycles.	El Andaloussi et al. (93)
Human BMDSC	QT-induced POF mice	Direct ovarian infusion	Follicle count and ovarian weight increase. Hormone restoration and pregnancy rates improvement.	Mohamed et al. (94)
Human BMDSC	QT-induced POR and POR mice	IV tail injection	Preovulatory follicle and MII oocytes increase. Fertility restoration, pregnancy rates and litter size increase. Apoptosis reduction, vascularization, and cellular proliferation increase. Ability of BMDCSs to migrate and infiltrate xenotransplanted human ovaries, promoting vascularization, follicle development and E2 secretion.	Herraiz et al. (95)

BMDSC, Bone marrow derived stem cells; POI, premature ovarian insufficiency; POF, premature ovarian failure; POR, poor ovarian responder; AFC, antral follicle count; AMH, Anti-Mullerian hormone; IVF, in vitro fertilization; IV, intravenous; E2, Estradiol; TNF-α, Tumor Necrosis Factor α; IGF-I, Insulin-Like Growth Factor 1; GCs, Granulose Cells.

In spite of the greater potential of MSCs, others SCs sources have been explored. Liu et al., described the potential of **human embryonic stem cell (ESCs)** to restore hormone levels and increase follicular count in mice, after the vein injection in vesicles, possibly by means of the apoptosis reduction (101). However, the use of ESCs, which may be obtained from the blastocysts inner cell mass is not exempt from ethical concerns.

In light of reported studies and advances in this field by using preclinical animal models, there are higher expectations regarding the use of MSCs, and especially BMDSCs to restore ovarian function in humans.

### Pilot Studies and Clinical Trials in POI Patients

The firsts clinical trials developed in humans using MSCs from bone marrow (BM) required iliac crest aspiration for cell collection followed by SCs isolation and *in vitro* culture procedures to reach clinically relevant cell numbers (**Tables 2.1** and **2.2**).

**Table 2.1:** Human studies involving bone marrow stem cell treatment for POR patients.

Regenerative factor	Study population	Administration method	Main findings	Limitations	Reference
BM-MSC	33 patients with idiopathic/other POF/POI and low ovarian reserves. Baseline characteristics not yet reported.	BM-MSCs into both ovaries *via* laparoscopy.	Not yet reported	Still ongoing	Al-Hendy et al. (NCT02696889)
BMDSC	17 POR patients (<40 years old). AMH = 1.9 ± 0.6 pM AFC = 4.0 ± 1.3	One ovarian artery by intraarterial catheterism	-81.3% POR improved AFC and AMH 2 weeks after treatment. - 33.3% treatment PR. - 5 pregnancies and 3 live births.	16% euploidy rate due to advanced maternal age was not ameliorated.	Herraiz et al. (102)

BM-MSC, bone marrow mesenchymal stem cells; BMDSC, bone marrow derived stem cells; POI, premature ovarian insufficiency; POF, premature ovarian failure; POR, poor ovarian responder; AFC, antral follicle count; AMH, anti-Mullerian hormone; FSH, follicle stimulating hormone; COS, controlled ovarian stimulation; IVF, in vitro fertilization; GSC-F, granulocyte colony-stimulating factor; ASCOT, autologous stem cell ovarian transplantation. Modified from Herraiz et al. (103).

**Table 2.2:** Human studies involving bone marrow stem cell treatment for POI and perimenopausal patients.

Regenerative factor	Study population	Administration method	Main findings	Limitations	Reference
BM-MSC	1 perimenopausal woman (45-year old). AMH 0.4 ng/ml AFC = 1	BM-MSCs into both ovaries *via* laparoscopy.	-AFC and AMH increased 8 weeks after treatment. -1 live birth.	POR similar to that reported for POI patients without treatment	Gupta et al. (17)
BM-MSC	10 women with idiopathic POI (26–33 years old). AMH <0.1 ng/ml; FSH = 58 mIU/ml	BM-MSCs into both ovaries *via* laparoscopy.	-Resumption of menses in 20% patients after 3 months. -10% treatment POR. -One pregnancy and a live birth in one patient showing endometrial regeneration.	POR similar to that reported for POI patients without treatment	Edessy et al. (104)
BM-MSC	30 patients with POF (18–40 years old). Baseline characteristics not reported.	Direct laparoscopic infusion into the ovarian stroma and catheterism into the ovarian artery of one side.	-86.7% POF patients improved hormone profile 4 weeks after treatment. -60% showed ovulation. -3 patients underwent IVF. -1 spontaneous pregnancy.	-AFC not reported or compared between ovaries. -IVF outcomes were not reported.	Gabr et al. (105)
BM-MSC	33 patients with idiopathic/other POF/POI and low ovarian reserves. Baseline characteristics not yet reported.	BM-MSCs into both ovaries *via* laparoscopy.	Not yet reported	Still ongoing	Al-Hendy et al. (NCT02696889)
BMDSC	20 POI patients (<39 years old). (10 patients included) Baseline characteristics not reported	One ovarian artery by intraarterial catheterism (ASCOT) (6 patients) and stem cells mobilization to peripheral blood by means of GSC-F (4 patients).	-Follicular development in both arms (90–140 days after treatments). -AFC increase in 50% of patients (GSC-F arm) and 66.6% of women (ASCOT arm). -Statistically significant FSH decrease is not observed, although FSH decreased was decreased. -In G-CSF arm: COS initiated in 2/4 women and 1 embryo vitrified. Embryo transfer was performed but pregnancy was not achieved -In the ASCOT arm: COS initiated in 4/6 women, 1 embryo vitrified and transferred, having an ongoing pregnancy. -Menses recovery in 40% of patients and climacteric symptoms decrease in 50% of women.	Still ongoing. Preliminary data from interim analysis reported (106)	Herraiz et al., (NCT03535480)

BM-MSC, bone marrow mesenchymal stem cells; BMDSC, bone marrow derived stem cells; POI, premature ovarian insufficiency; POF, premature ovarian failure; POR, poor ovarian responder; AFC, antral follicle count; AMH, anti-Mullerian hormone; FSH, follicle stimulating hormone; COS, controlled ovarian stimulation; IVF, in vitro fertilization; GSC-F, granulocyte colony-stimulating factor; ASCOT, autologous stem cell ovarian transplantation. Modified from Herraiz et al. (106).

Gupta et al. (17) published a live birth in a postmenopausal woman (45 years old) underwent this technique and IVF treatment. They injected cells in both ovaries by laparoscopy. This means to expose the patient to two different invasive procedures: first the iliac crest aspiration, and second the laparoscopy. Edessy’s group followed the same technique, in 10 POI younger women (26–33 years old) with positive results showing a return of menses in two patients and one ongoing pregnancy, with one live birth (104). Gabr et al. (105) later applied this same method in 30 POI women (18–40 years old). This study, instead, had two branches: one arm received these cells by direct ovarian injection through laparoscopy, while the second arm had cells injected through de ovarian artery. One spontaneous pregnancy was obtained. Al Hendy and colleagues are carrying out similar studies as the above described in POR and POI patients, after their promising results in animals, but their investigations are still ongoing.

We recently described that infusion of BMDSC promotes human and mouse follicular growth by increasing ovarian vascularization, stromal cell proliferation, and reducing cell death (95). Based on this information, a prospective pilot study in 17 POR women was developed by our group to evaluate the effects of autologous stem cell ovarian transplant (ASCOT) on ovarian reserve (102). ASCOT improved ovarian function biomarkers (AMH and AFC) in 81.3% of women and a total of six pregnancies and three healthy babies were achieved. ASCOT improved follicle and oocyte quantity enabling pregnancy in POR women previously limited to oocyte donation. In the context of ovarian tissue, stem cell paracrine actions should be evaluated for their capacity to activate the pre-existing quiescent follicles based on the ability of BMDSCs to produce and secrete a broad variety of growth factors involved in follicular growth, angiogenesis, viability, and ovarian response to Controlled Ovarian Stimulation (COS) (107). In fact, our results suggest that ASCOT optimized the growth of existing follicles, mediated the presence of specific stem cell secreted factors such as FGF-2 and THSP-1 within aphaeresis. Based on that, a randomized pilot study (NCT03535480) has been designed with 20 POI women younger than 39 (106). Patients will be randomized to the ASCOT or only stem cell mobilization based on the ability of ovarian niche to attract undifferentiated cells from BM in a process known as “homing’’ (89).

It is important to highlight that many other stem cell origins are also being tested worldwide in several RCT involving POI women. Nevertheless, most of them are still ongoing and therefore results have not been yet reported (**Table 3**).

**Table 3:** Registered Randomized Clinical Trials involving different sources of SCs—apart from Bone Marrow Derived Stem Cells—for POF/POI patients.

Regenerative factor	Study population	Inclusion criteria	Number of clinical trial	Status
Human umbilical cord mesenchymal stem cells (hucMSCs)	12 patients with POF	-Diagnostic criteria of ESRHE -No hormonotherapy within 3 months	NCT03816852	Suspended
Human umbilical cord mesenchymal stem cells (hUCMSCs) and human cord blood mononuclear cells (hCBMNCs)	40 patients with POF	-Age: 18–39 -Clinical diagnosis of POF -Currently receiving Hormone Replacement Therapy	NCT01742533	Unknown
Human Embryonic Stem Cell Derived Mesenchymal Stem Cell (MSC)-Like Cells	18 patients with POF	-Age >40 -Have established regular menstrual cycle, oligomenorrhea/amenorrhea ≥4 months -FSH >25 IU/ml -Bilateral ovaries visible by ultrasound -Fertility requirement and sperms in couple	NCT03877471	Recruiting
Autologous very small embryonic-like stem cells (VSELs)	Estimated POF population not shown	-Clinical diagnosis of POF -Abnormal sex hormone levels	NCT03985462	Withdrawn
Human Adipose Derived Mesenchymal Stromal Cells	9 patients with POF	-Age: 20–39 -FSH >20	NCT02603744	Unknown
Human Adipose derived stem cells (ADSC)	4 patients with POF	-Age: 20–39 -Clinical diagnosis of POF -Lack of response to drug treatment -Willing to receive follow-up -Willing to conceive a baby	NCT01853501	Unknown
Human Umbilical Cord Mesenchymal Stem Cells (hUC-MSCs)	320 patients with POF	-Age: 20–40 -Clinical diagnosis of POF -Fertility requirement and sperms in couple	NCT03033277	Unknown
Human Umbilical Cord-derived Mesenchymal Stem Cells (hUC-MSCs)	23 patients with POF	-Age: 20–39 -Clinical diagnosis of POF -Lack of response to drug treatment	NCT02644447	Completed
Ovarian Stem Cells	11 patients with POF	-Age: 20–39 -Clinical diagnosis of POF, POI, or DOR -Early follicular phase FSH >15 IU/L -AMH <0.16 ng/ml or below the level of detection for the assay used -Undergoing ovarian biopsy by laparoscopy or clinically indicated abdominal surgery that provides access to the ovaries -Early follicular phase FSH >15 IU/L -AMH <0.16 ng/ml or below the level of detection for the assay used	NCT01702935	Completed

POF, premature ovarian failure; ESRHE, European Society of Human Reproduction and Embryology; FSH, follicle stimulating hormone; POI, premature ovarian insufficiency; DOR, diminished-ovarian reserve.

## Proposed Mechanisms for Stem Cell Therapy

As it has been described, different studies showed that BMDSCs are effective for POI treatment in animals (and present promising results in humans) (103). To understand the underlying mechanisms would allow us to optimize these strategies and to find the optimal cohort of patients who will be benefited.

**Figure 2:**
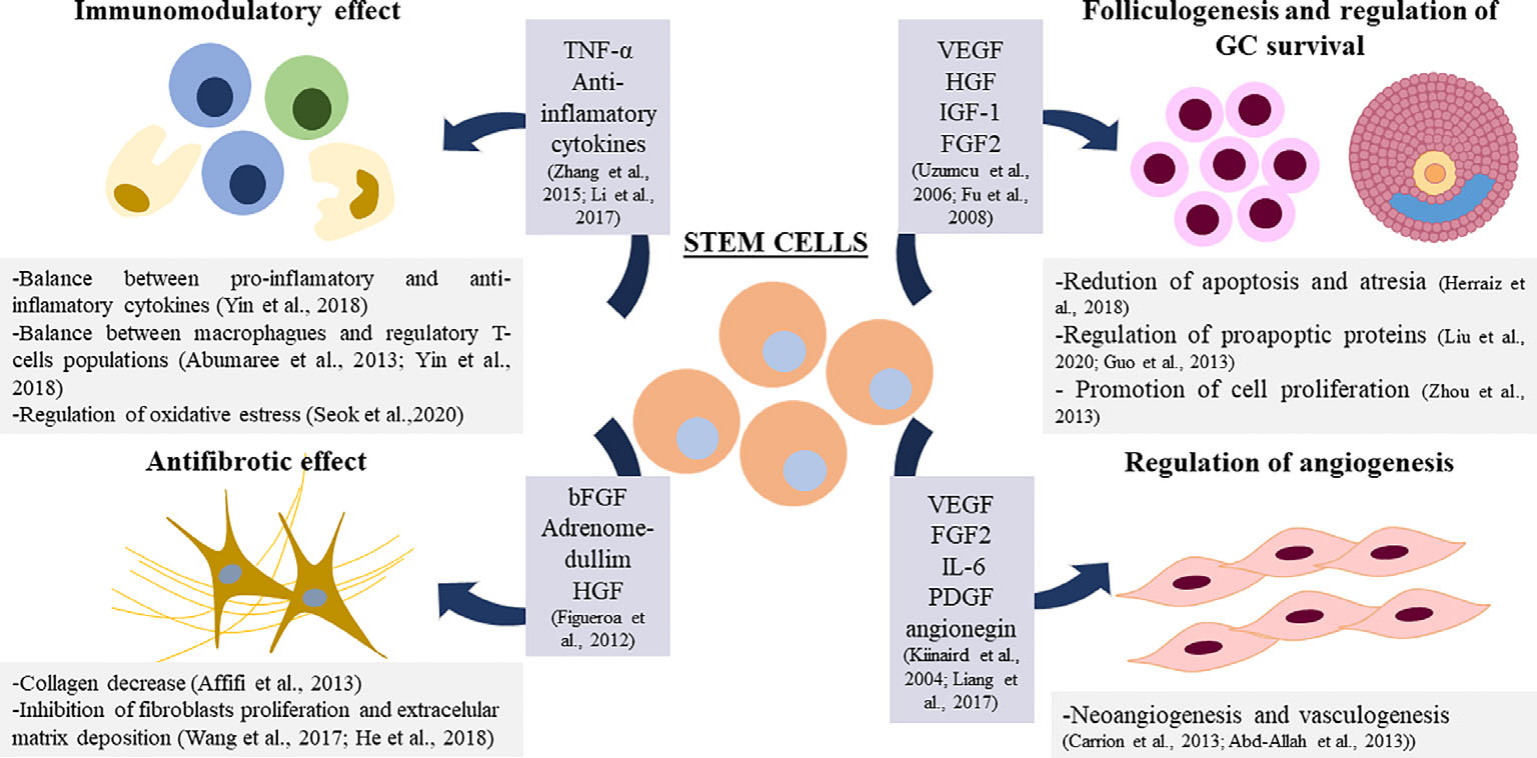
Proposed mechanisms for Stem Cell Therapy in ovarian damage. TNF-α: Tumor Necrosis Factor α, VEGF: Vascular Endothelial Growth Factor, HGF: Hepatocyte Growth Factor, IGF-1: Insuline Like Growth Factor 1, bFGF: Basic Fibrolast Growth factor, FGF2: Fibrolast Growth Factor 2, IL-6: Interkuline 6, PDGF: Platelet Derived Growth Factor.

Overall, SCs show the ability to act in a paracrine manner thanks to the secretion of soluble factors and chemokines (75, 79, 81, 91). Paracrine action could help to restore damaged tissue, in this case the ovarian niche, by regulating different vital processes in this microenvironment. In this context, different studies show the involvement of BMDSCs in the regulation of angiogenesis, apoptosis, the regulation of the immune system, and fibrosis in the ovary (108) (**Figure 2**).

In the context of angiogenesis regulation in the ovary mediated by BMDSCs, the increase of vascularization in the ovarian niche, improve the healing process that occurs in a cyclic manner, and it may be beneficial for ovarian recovery (91). It has been reported that factors produced by these cells such as Vascular Endothelial Growth Factor (VEGF), Fibroblast Growth Factor-2 (FGF2) and Interleukine-6 (IL-6) promote arteriogenesis *in vitro* and *in vivo* (109). BMDSCs have been shown to promote angiogenesis *in vitro* through the α5 β1 receptor (110) and through Platelet Derived Growth Factor (PDGF) (111). In ovarian tissue, angiogenin produced by BMDSCs has been reported to play a positive role in angiogenesis after transplantation (112).

Regarding the antiapoptotic-promotion property of BMDSCs in GCs in the ovary (72, 81, 87, 95, 101), it has been reported that the coculture with BMDSCs decreases the levels of the proapoptotic proteins P21 and BAX and increase the levels of the proto-oncogene *c-myc* in GCs (100, 101). It has been observed that different cytokines present in the BMDSCs culture medium—VEGF, HGF, IGF-1—are able to decrease apoptosis of granulosa cells *in vitro* (113) and *in vivo* (87) and promote their proliferation (114); which suggests that the secretion of these growth factors may be underlying the apoptosis decrease found after BMDSCs in the ovary.

The immunomodulatory effects of BMDSCs have been tested *in vitro* and *in vivo* in different diseases (115, 116). The regulation of the balance between different populations of immune cells or between pro-inflammatory and anti-inflammatory cytokines mediated by BMDSCs could underlie this immunomodulatory effect (108). MSCs have been reported to have immunoregulatory properties in the ovarian niche by regulating populations of macrophages, regulatory T lymphocytes, and associated cytokines (117, 118). TNF-alpha has also been associated with the immunoregulatory function of human MSCs in the ovary (119). MSCs have been reported to reduce SOD dismutase in the ovary after transplantation, suggesting that the recovery of the ovarian niche could also be due to a regulation of oxidative stress in this microenvironment (108, 120).

In relation to fibrosis decrease, after BMDSCs transplantation, a decrease in collagen levels has been observed (121), suggesting that the mechanism of action of these cells in the recovery of ovarian damage could also involve an antifibrotic effect (79). In fact, BMSCs may inhibit fibroblasts proliferation and decrease the level of extracellular matrix deposition (108).This antifibrotic effect has been associated with certain soluble factors such as HGF, adrenomedullim and Basic Fibroblast Growth Factor (bFGF) (122).

As it is said, the paracrine action of BMDSCs in the ovarian niche has been demonstrated by injecting only soluble factors from the culture medium of the SCs, obtaining similar results as with SCs transplant (79). In fact, the ASCOT clinical trial in POR women highlighted the fact that aphaeresis provided relevant components for success, including specific BMDSC-secreted soluble factors, which acting in a paracrine manner promote growth of the already existing residual follicles in impaired ovaries. This study found that positive response was not limited to the injected ovary, as circulating SCs during the mobilization phase also reached the non-injected ovary producing an increase in the AFC in both sides (102). Furthermore, there are recent reports of nervous tissue rejuvenation and repair by injection of young growth factor enriched plasma, umbilical cord blood plasma, and plasma specific proteins into damaged and aged organisms (123–125).

This fact opens up new possibilities to combat damage in ovarian tissue such as the injection of soluble factors or platelet-rich plasma (PRP).

## The PRP Approach

PRP injection has been used for years in several fields of Medicine (orthopedics, sports Medicine, aesthetics, etc.) but in the context of assisted reproductive techniques (ART), intraovarian injection of autologous PRP has been recently proposed as an alternative to restore ovarian function in POI women (**Tables 4.1** and **4.2**). This approach is also based on the paracrine signaling, as PRP is a concentrate composed by platelet-enclosed growth factors, which could promote tissue healing, angiogenesis and cell growth (132, 133). Pantos et al. (129) introduced by first time this new approach without the direct use of SCs to reactivate folliculogenesis in perimenopausal women. In this study, the ovaries of eight perimenopausal women of advanced maternal age (41–49 years old) were infused with platelet-rich plasma by transvaginal ultrasound-guided injection. Treatment resulted in restoration of menses, with presence of ovarian follicles that allowed oocyte retrieval after IVF treatment in all patients and cryopreservation of 1.50 ± 0.71 embryos. However, a limitation of the study is that it only included eight women and did not document their previous ovarian reserves. These effects might be due to an increased in ovarian vascularization, with a key role in ovarian function as well as in promoting follicle development increasing follicular cell proliferation and survival (95). After this first report, ovarian and endocrine positive effects and live births have been also reported in several series of patients with impaired ovarian function such as POR (127) and POI women (130, 134).

**Table 4.1:** Human studies involving PRP treatment for POR patients.

Study population	Infertility history	PRP preparation	Administration method	Main findings	Reference
4 patients with DOR (38–46 years old) AMH = 0.38 ng/ml ± 0.38 FSH = 13.6 mIU/ml AFC = 4 ± 0.8	Infertility duration 60 ± 25 months	Centrifugation and activation with calcium gluconate	Transvaginal ultrasound-guided ovarian stroma injection.	-AMH increase or/and FSH decrease in all cases -Oocytes retrieval (5.3 ± 1.3 MII oocytes) in all cases. -IVF occurred range 59–110 days after treatment. -At least one cryopreserved blastocyst for each patient.	Sills et al. (126)
					
23 PORs (34–40 years old) AMH <0.5–1.1 ng/ml	Infertility duration 0.5 ± 3.77 years	Blood Transfusion Organization standard method	Transvaginal ultrasound-guided ovarian injection	-Oocyte retrieval increase (2.1 *vs* 0.64 before treatment). -2 spontaneous conceptions. -3 live births.	Farimani et al. (127)
120 patients. -30 POR patients (38.40 ± 2.01) AMH = 0.66 ± 0.20 ng/ml; FSH = 10.71 ± 1.62 IU/ml; AFC = 2.63 ± 0.93*	Infertility duration: -5.82 ± 1.02 years (POR group)	RegenACR- C Kit	Transvaginal ultrasound-guided intramedullar ovarian injection	-POR patients: AMH and AFC increased and FSH decrease two menstrual cycles after treatments. 14 pregnancies and 12 live births.	Sfakianoudis et al. (128)

POR, poor ovarian responder; DOR, diminished ovarian reserve; AFC, antral follicle count; AMH, anti-mullerian hormone; FSH, follicle-stimulation hormone; COS, controlled ovarian stimulation; *reported only for those women with positive response after PRP.

**Table 4.2:** Human studies involving PRP treatment for POI and perimenopausal and menopausal patients.

Study population	Infertility history	PRP preparation	Administration method	Main findings	Reference
8 perimenopausal women with idiopathic POI (41–49 years old). Baseline characteristics not reported.	Amenorrhea duration 4.88 ± 1.13 months	RegenACR- C Kit	Transvaginal ultrasound-guided ovarian injection.	-Menstrual cycle restoration 1–3 months after treatment. -Follicle development and oocyte retrievals in all cases, (1.50 ± 0.71 MII oocytes.) -1.50 ± 0.71 resultant embryos -Cryopreserved transfer has been performed.	Pantos et al. (129)
2 women with POF (47 and 27 years old) and a menopausal woman (46 years old). AMH 0.06–0.17 ng/ml; FSH 46.5–119 mIU/ml; AFC = 0	Amenorrhea duration 12 months (menopausal patient), not reported (POF patients)	RegenACR- C Kit	Transvaginal ultrasound-guided ovarian injection	-Menstrual cycle restoration in all cases 1–2 months after treatment. -AMH increase or/and FSH decrease in all cases -Pregnancy in natural conception through natural conception 2–6 months after treatment.	Pantos et al. (130)
1 woman with premature menopause (40 years old) AMH = 0.02 ng/ml; FSH = 149 mIU/ml	Amenorrhea duration 19 months	RegenACR- C Kit	Transvaginal ultrasound-guided intramedullar ovarian injection	-Menstrual cycle restoration 6 weeks after treatment. -FSH decrease and slightly AMH increase. -Biochemical pregnancy, resulting in a spontaneous abortion at the 5th week of pregnancy.	Sfakianoudis et al. (126)
120 patients. -30 patients with POI (35.9 ± 1.9 years old); AMH = 0.18 ± 0.04 ng/ml; FSH = 40.611 ± 6.05 IU/ml; AFC = 0* -30 perimenopausal women (43.4 ± 1.4 years old). AMH = 0.96 ± 0.28 ng/ml; FSH = 18.51 ± 2.62 IU/ml; AFC = 1.54 ± 0.51* -30 Menopausal women (48.8 ± 1.6 years old). AMH= 0.13± 0.03 ng/ml; FSH= 80.27 **±** 5.03 IU/ml; AFC=0 *	Amenorrhea duration: -16 ± 2.42 months (POI group) -15.69 ± 1.75 months (Menopausal group)	RegenACR- C Kit	Transvaginal ultrasound-guided intramedullar ovarian injection	-POI patients: menses recovery and FSH increase in 60% of patients. 3 pregnancies and 3 live births. -Perimenopausal patients: menses recovery and FSH increase in 80% of patients. 4 natural pregnancies and 3 live births. -Menopausal patients: menses recovery and FSH increase in 43.3% of patients. 1 pregnancy and 1 live birth.	Sfakianoudis et al. (128)
311 patients with POI (34.6 ± 4.0 years old) AMH = 0.01–0.82 ng/ml FSH = 25–155 mIU/ml** AFC = 1.26 ± 0.8	Infertility duration 6.8 ± 4.9 years	Centrifugation and T-lab autologous platelet-rich plasma kit (T-Biotechnology Laboratory)	Transvaginal ultrasound-guided intramedullar underneath ovarian cortex injection	-Spontaneous pregnancy in 7.4% of patients (69.6% achieved live birth). -AMH and AFC increased after treatment. FSH increase not observed. -Antral follicle observation and COS initiation in 64.6% of patients. 40.8% of these patients achieved at least one blastocyst. -22.8% of stimulated patients achieved a pregnancy after transfer.	Cakiroglu et al. (131)

POI, premature ovarian insufficiency; POF, premature ovarian failure; AFC, antral follicle count; AMH, anti-Mullerian hormone; FSH, follicle-stimulation hormone; COS, controlled ovarian stimulation.

*Reported for women with positive response after PRP; ** Reported only for those women achieving pregnancy after PRP.

Sills et al. (126) showed, in a study population of aged women (42 ± 4 years; infertility duration 60 ± 25) with diminished ovarian reserves, that intraovarian administration of PRP was able to induce an increase in serum AMH and a decrease in serum FSH, sufficient to permit oocyte retrieval (5.3 ± 1.3 MII) and blastocyst cryopreservation in all recruited patients 2 months after treatment.

The firsts controlled clinical trials involving a relevant number of patients with different and properly characterized ovarian phenotypes have been published in the last year. Sfakianoudis et al. (128) reported four pilot studies on POR, POI, perimenopausal, and menopausal women with a total of 120 participants recruited (n = 30 each). In the case of POR women (38.4 ± 2.0 y.o.), they found that PRP injection was able to improve ovarian reserve biomarkers, as AMH levels as well as AFC increased in the first and second menstrual and remained stable in the third while FSH and LH levels were reduced in the first menstrual cycle and remained stable. The main ICSI cycle outcomes were increased, especially the number of oocytes retrieved and the number of MII and embryos, all together with a reduction of the cancelation rate, a main concern in POR women. Overall, the reported clinical pregnancy rate for POR was 46.6% (14 out of 30 women) with 12 participants having a live birth.

For the other three pilot studies included, the reported primary outcomes were different according to the diagnosis of the recruited patients, as for menopause and POI women menses recovery and FSH levels became a principal result. In the POI population (35.9 ± 1.9 y.o.), they observed that 18 women (60%) positively responded to PRP treatment when considered as menstrual cycle restoration and reduced FSH levels, with a total of three pregnancies and three live births (PR:10%). These results slightly improved in the perimenopausal women (43.4 ± 1.4 y.o.), where 24 women (80%) positively responded to PRP treatment. For these women menstrual cycle regulation as well as FSH level reduction was observed having four natural conceptions and three live births (PR:13.3%). Finally, in the menopausal group (48.8 ± 1.6 y.o.), 13 women (43.3%) positively responded to PRP treatment with one pregnancy and one live birth (1%).

To date, the largest study has been developed by Cakiroglu et al. (131), in a population of 311 women (34.8 ± 4.3 y.o.) with POI diagnosis based on the ESHRE criteria. After intraovarian injection of autologous PRP, the 7.4% of POI women (23/311) achieved a spontaneous pregnancy one or two menstrual cycles after treatment with 7 miscarriages and 16 live births reported. From the remaining patients, development of at least one antral follicle was noticed in 201 allowing the initiation of controlled ovarian stimulation from the second to the sixth menstrual cycles after intervention, although oocyte retrieval was only achieved in 130 and MII-oocytes obtained in 93 women. The 40.8% of stimulated women obtained at least one cleavage stage embryo scored as A/B according to morphological criteria. To date, only 57 of these patients underwent embryo transfer (both fresh or frozen) as the remaining ones having embryos decided to cryopreserve them for a later transfer. A total of 13 achieved a pregnancy after ET (22.8%) although 4 experienced a miscarriage. Nevertheless, FSH levels did not improve after treatment when compared to previous values, although AMH and AFC increased. Overall, this study reported a total of 36 pregnancies in 311 women (11.5%PR) and 8% of live birth rate or sustained implantation, which although opening a new path for the management of POI women. It is relevant for the overall evaluation of these rates to highlight that at the moment of publication several patients still had their embryos cryopreserved for future transfer.

All together, the studies evaluating PRP ovarian injection are encouraging as they open a new path to a clinical alternative more easily applied than the stem cell based therapies as ovarian injection is performed in a similar intervention to oocytes collection. Nevertheless, their results should be evaluated with caution as for now there are no experimental studies evaluating the wide spectrum of PRP effects, duration, and mechanism in the ovarian tissue, and the reported human studies lack from an adequate control group to properly establish the efficiency of the technique. Thus, results of placebo in double blinded randomized clinical trials should be obtained and carefully evaluated before proposing PRP as a routine treatment for POI and DOR patients in ART clinics. Furthermore, it is important to bear in mind that POI pregnancy rates across studies ranged from 2.2 to 14.2% and spontaneous resumption of ovarian function occurs in 25% of patients, and primordial and pre-antral follicles are frequently found in ovarian biopsies from women diagnosed as having POI (8).

## Conclusions

This field of investigation opens new opportunities for ovarian rescue in women with impaired ovarian reserve, such as POR and POI patients, by different strategies focused on the rescue of already existing follicles. These approaches include the inhibition of molecular pathways by IVA and tissue mechanical fragmentation, stem cell administration, and PRP ovarian injection. Although heterogeneous, all the techniques have a common characteristic, to promote growth of follicular cells by activating different paracrine signaling mechanisms. This finding is of paramount relevance for the future design of feasible and less invasive clinical options. Nevertheless, these proposals should be previously supported by comprehensive experimental and mechanistic studies. The inclusion of a proper control group should be mandatory in future randomized clinical trials for a realistic evaluation of the technique’s efficacy in selected group of patients.

## Author Contributions

AP has performed a bibliography search and analysis and she has written the manuscript. JG-V has critically revised the manuscript, and he has coordinated the study. SH has performed a literature search and analysis, she has written and critically revised the manuscript, and she has coordinated the study. All authors contributed to the article and approved the submitted version.

## Funding

This work was partially supported by grants FPU18/02904 and CP19/00141 from the Spanish Ministry of Science, Innovation and Universities for the participation of AP and SH, respectively.

## Conflict of Interest

The authors declare that the research was conducted in the absence of any commercial or financial relationships that could be construed as a potential conflict of interest.
